# The long non-coding RNA TAZ-AS202 promotes lung cancer progression via regulation of the E2F1 transcription factor and activation of Ephrin signaling

**DOI:** 10.1038/s41419-023-06277-y

**Published:** 2023-11-18

**Authors:** Giulia Gobbi, Alessandra Grieco, Federica Torricelli, Elisabetta Sauta, Giacomo Santandrea, Eleonora Zanetti, Valentina Fantini, Francesca Reggiani, Silvia Strocchi, Massimiliano Paci, Manik Vohra, Srinivas Vinod Saladi, Davide Carlo Ambrosetti, Alessia Ciarrocchi, Valentina Sancisi

**Affiliations:** 1 Laboratory of Translational Research, Azienda USL-IRCCS di Reggio Emilia, Reggio Emilia, Italy; 2 IRCCS Humanitas Clinical and Research Center, Milan, Italy; 3 Pathology Unit, Azienda USL-IRCCS di Reggio Emilia, Reggio Emilia, Italy; 4 Thoracic Surgery Unit, Azienda USL-IRCCS di Reggio Emilia, Reggio Emilia, Italy; 5 Department of Otolaryngology, Head and Neck Surgery, Massachusetts Eye and Ear Infirmary, Boston, USA; 6 Harvard Medical School, Boston, MA 02114 USA; 7 Broad Institute of MIT and Harvard, Cambridge, MA 02142 USA; 8 Department of Pharmacy and Biotechnologies (FaBit), University of Bologna, Bologna, Italy

**Keywords:** Non-small-cell lung cancer, Long non-coding RNAs, Transcription

## Abstract

Long non-coding RNAs (lncRNAs) are transcripts without coding potential that are pervasively expressed from the genome and have been increasingly reported to play crucial roles in all aspects of cell biology. They have been also heavily implicated in cancer development and progression, with both oncogenic and tumor suppressor functions. In this work, we identified and characterized a novel lncRNA, TAZ-AS202, expressed from the TAZ genomic locus and exerting pro-oncogenic functions in non-small cell lung cancer. TAZ-AS202 expression is under the control of YAP/TAZ-containing transcriptional complexes. We demonstrated that TAZ-AS202 is overexpressed in lung cancer tissue, compared with surrounding lung epithelium. In lung cancer cell lines TAZ-AS202 promotes cell migration and cell invasion. TAZ-AS202 regulates the expression of a set of genes belonging to cancer-associated pathways, including WNT and EPH-Ephrin signaling. The molecular mechanism underlying TAZ-AS202 function does not involve change of TAZ expression or activity, but increases the protein level of the transcription factor E2F1, which in turn regulates the expression of a large set of target genes, including the EPHB2 receptor. Notably, the silencing of both E2F1 and EPHB2 recapitulates TAZ-AS202 silencing cellular phenotype, indicating that they are essential mediators of its activity. Overall, this work unveiled a new regulatory mechanism that, by increasing E2F1 protein, modifies the non-small cell lung cancer cells transcriptional program, leading to enhanced aggressiveness features. The TAZ-AS202/E2F1/EPHB2 axis may be the target for new therapeutic strategies.

## Background

Lung cancer is an aggressive disease causing 1.8 million deaths every year [1]. Based on cellular morphology, it is classified into two main histotypes of which Non-Small Cell Lung Cancer (NSCLC) represents approximately 80% of cases. Despite the relevant recent progress made in diagnosis and therapy, the overall survival rate remains low, with only 17% of patients alive five years after diagnosis [1, 2]. Thus, defining novel molecular mechanisms underlying the pathogenesis of this disease is fundamental to improve patients’ survival.

In recent years, a great attention has been given to the role of long non-coding RNAs (lncRNAs) in regulating cell proliferation, migration, invasion, apoptosis and differentiation, processes relevant in cell physiology but often deregulated in cancer cells. LncRNAs have been shown to influence many biological processes, through a variety of molecular mechanisms, such as regulation of expression of neighboring genes in cis, re-organization of nuclear architecture, regulation of mRNAs, miRNAs and proteins abundance and/or function in trans [3]. Given their ability to control different and sometimes contrasting cancer properties, lncRNAs, like protein-coding genes, can act either as oncogenes or as tumor suppressors [3].

YAP and TAZ are paralogue transcriptional regulators that showed a crucial role in cancer progression, being able to integrate mechanical, metabolic and signaling inputs to enhance cell growth and malignant properties [4, 5]. The Hippo pathway negatively regulates YAP/TAZ abundance as well as their nuclear localization. In NSCLC, YAP preferentially regulates cell division and cell cycle progression, whereas TAZ mostly regulates migration and extracellular matrix organization [6]. Both YAP and TAZ have been implicated in cancer cells resistance to different anticancer agents [7]. Neither of these factors contains a DNA binding domain, relying for their activity on the binding to other transcription factors, such as TEADs [4, 5].

E2F1 is a transcription factor playing a key role in cell cycle progression and apoptosis regulation [8]. In lung carcinoma, E2F1 is overexpressed and is a pro-oncogenic factor, controlling different malignant properties of cancer cells beside proliferation, such as migration, invasion, epithelial-mesenchymal transition and metastasis [9–13]. E2F1 expression and activity is regulated by multiple mechanisms, acting both at transcriptional and post-transcriptional level [14–16].

Different lncRNAs have been shown to modulate both YAZ/TAZ and E2F1 expression and activities, holding the capacity to foster or restrain the cancer-promoting role of these transcription factors [17–20].

In this work, we identify and characterize a novel lncRNA, TAZ-AS202, which is transcribed from the TAZ genomic locus and is overexpressed in lung adenocarcinoma compared with healthy lung tissue. TAZ-AS202 enhances aggressive properties of NSCLC cell lines, such as migration and invasion, by regulating a set of genes belonging to cancer-associated pathways, including WNT and EPH-Ephrin signaling. Mechanistically, TAZ-AS202 does not affect TAZ or YAP expression or localization, but instead regulates the E2F1 protein, which in turn transcriptionally regulates EPHB2 and a large set of genes affected by TAZ-AS202 silencing. The downregulation of E2F1 or EPHB2 restrain cell invasion and migration, recapitulating the phenotype observed upon TAZ-AS202 silencing. Overall, our work uncovers a novel axis promoting NSCLC aggressiveness and progression.

## Methods

### Cell cultures and treatments

A549 and NCI-H23 cell lines were obtained from Dr. Giovanna Damia (IRCCS-Istituto di Ricerche Farmacologiche Mario Negri, Milan, Italy). All cell lines were authenticated by SNP profiling at Multiplexion GmbH. Cells were tested for mycoplasma infection with a monthly schedule. All cell lines were grown at 37 °C/5% CO2 in RPMI with 10% fetal bovine serum and antibiotics.

### siRNA transfection

YAP, TAZ, TAZ-AS202 #1 TAZ-AS202 #2, TAZ-AS203, EPHB2 and E2F1 Silencer Select RNAi (30 nM) and control oligos (Life Technologies) were transfected using RNAiMax Lipofectamine (Life Technologies). For further details on each siRNA and control oligos, see Supplementary Table 1. If not otherwise specified, cells were harvested 48 h after transfection for qRT-PCR, Western blot and/or ChIP analysis.

### Proliferation assay

24 h after siRNA transfection, 3000 A549 cells or 4000 NCI-H23 cells were seeded in triplicate in a 96-well culture plate. Phase area of confluence, normalized to T0 (time 0), was analyzed 24, 48 and 72 h after the seeding using Incucyte S3 live cell imaging system (Sartorius).

### Migration assay

24 h after siRNA transfection against TAZ, TAZ-AS202 #1, TAZ-AS202 #2, EPHB2 or control oligos, 1 million of A549 and NCI-H23 cells were seeded in a 6-well culture plate. The day after, cells were treated with mitomycin (Sigma Aldrich, Milan, Italy) at the concentration of 2 µg/ml for A549 and 1 µg/ml for NCI-H23, for 1 hour and 30 min. 48 h after siRNA transfection against E2F1, the same number of cells were seeded in a 6-well culture plate. The day after, cells were treated with the same concentration of mitomycin. Cell medium was replaced with normal complete culture medium. Scratches were applied using a pipette tip. Healing areas were captured at 0, 19, 26, 48 and 54 h after the scratch for siRNA against TAZ, TAZ-AS202 and EPHB2, at 0, 19, 26 and 44 h after the scratch for siRNA against E2F1, using a Nikon Ti-E inverted microscope (Nikon Instruments, Florence, Italy). Three images per condition were taken. The area of the scratch was calculated at each time point using ImageJ software and each time point was normalized on the specific area of T0.

### Invasion assay

48 h after siRNA transfection against TAZ, TAZ-AS202 #1, TAZ-AS202 #2, EPHB2 or control oligos, 3 × 10^4^ A549 cells and 4 × 10^4^ NCI-H23 cells were seeded in a Matrigel Invasion Chamber or control chambers (CT insert) (BD Biosciences, San Jose, CA) in triplicate. For E2F1 silencing, 72 h after siRNA transfection, the same number of cells were seeded. Complete medium containing 10% FBS was used as chemo-attractant. The day after, invading cells were fixed with methanol, stained with crystal violet and pictures were obtained using a Nikon Ti-E inverted microscope. At least four fields for each well were captured and invading cells were manually counted. To obtain the graph, we divided the cells in the Matrigel Invasion Chamber with the cells in the control insert for each condition.

### Western blot

Western blot analysis was performed as previously described [21]. For EPHB2 detection, the following RIPA buffer was used for cells lysis: NaCl 150 mM, EDTA 5 mM, Tris pH8 50 mM, NP-40 1%, Na-deoxycolate 0.5%, SDS 0.1%. For the detection of all the other proteins, Passive lysis buffer 5X (Promega) was used for cells lysis. Each buffer was supplemented with protease inhibitor cocktail (bimake.com, Munich, Germany). For further details on primary and secondary antibodies, see Supplementary Table 1.

### Nuclear and cytosolic extracts preparation

For cytoplasmic and nuclear extract on not-treated cells, 8 × 10^5^ A549 or NCI-H23 cells were seeded in two T25 culture flask and cells were harvested 48 h after. For cytosolic and nuclear extract on treated cells, 8 × 10^5^ A549 or NCI-H23 cells were transfected with specific siRNA and cells were harvested 48 h after transfection. For total lysate, Passive lysis buffer 5X (Promega) was used. For the cytoplasmic lysis, the following buffer was used for A549 cells: 10 mM Hepes pH 7.9, 1.5 mM MgCl2, 100 mM KCl and 0.05% NP-40. For the cytoplasmic lysis, the following buffer was used for NCI-H23 cells: 10 mM Hepes pH 7.9, 1.5 mM MgCl2, 100 mM KCl and 0.025% NP-40. Nuclei were washed twice with the following buffer: 10 mM Hepes pH 7.9, 1.5 mM MgCl2, 100 mM KCl. For nuclear extraction, the following buffer was used: 20 mM Hepes pH 7.9, 25% Glycerol, 0.42 M NaCl, 1.5 mM MgCl2, 0.2 mM EDTA. Each solution was supplemented with Protease Inhibitor Cocktail (Bimake.com, Munich, Germany).

### RNA extraction and qRT-PCR analysis

Total RNA was extracted and purified with RNAesy Mini kit (Qiagen, Milan, Italy). RNA was quantified with Nanodrop 2000/2000c Spectrophotometer (Thermo Fisher Scientific, Monza, Italy) and 500/1000 ng RNA was retrotranscribed using iScript cDNA kit (Biorad, Segrate, Italy). Quantitative real time PCR (qRT-PCR) was conducted using Sso Fast EvaGreen Super Mix (Biorad) in the CFX96 Real Time PCR Detection System (Biorad), as previously described [22]. See Supplementary Table 1 for qRT-PCR primers. RPS7 was used as reference gene for siRNA E2F1 vs siCT conditions, Cyclophilin A was used as reference gene for all other conditions.

### RNA-sequencing analysis

RNA-seq was performed on A549 cells transfected with siRNA against TAZ, TAZ-AS202 #1 and control oligos in two independent biological replicates. For each experimental condition, cell pellets were collected 48 h after transfection. The total RNA was extracted and the downregulation of TAZ, TAZ-AS202 was verified by qRT-PCR. Samples were quantified at Qubit (Thermo Fisher Scientific, Milan, Italy) and loaded on Bioanalyzer-RNA 6000 nano kit (Agilent Technologies, Santa Clara, California, USA) for purity and quality assessment. Libraries were prepared starting from 500 ng RNA, using TruSeq Stranded total RNA kit (Illumina, San Diego, California, USA). Next generation sequencing was conducted by NextSeq 500 platform (Illumina, San Diego, California, USA) on high-output cartridge (2 × 75) and a minimum of 30 million of reads for each replicate was expected. Sequencing quality was assessed using FastQC v0.11.8 software (www.bioinformatics.babraham.ac.uk/projects/fastqc/), showing on average a Phred score per base >34 in each sample. Raw sequences were then aligned to the human reference transcriptome (GRCh38, Gencode release 30) using STAR version 2.7 and gene abundances were estimated with RSEM algorithm (v1.3.1). Differential expression analysis was performed using DESeq2 R package, considering a False Discovery Rate (FDR) of 5% and excluding genes with low read counts. Significant genes underwent to enrichment analysis, performed on Reactome pathways databases via enrichR package, using a significance threshold of 0.05 on *p*-value adjusted by Benjamini-Hochberg correction for multiple testing.

### Chromatin immunoprecipitation

The chromatin immunoprecipitation (ChIP) assay for YAP, TAZ and TEAD4 was performed on A549 cells as described previously [23]. Briefly, A549 cells were crosslinked with 2 mM EGS (ethylene glycol bis (succinimidylsuccinate)) followed by 1% formaldehyde. The cross-linked cell lines were quenched with 125 mM glycine. The fixed cells were lysed in RIPA buffer for 3–4 h followed by sonication for 8 min, 5 times each cycle in a bioruptor (Diagenode). Soluble chromatin was immunoprecipitated with 4 μg of YAP1, TAZ, TEAD4, or IgG antibody overnight followed by incubation with calibrated protein G beads for 3 h. Washes were performed with low salt (150 mM KCl) and high salt buffers (250 mM KCl). Reverse crosslinking was performed for 16 h and the precipitated DNA was purified by DNA Clean & Concentrator-5 Kit (Zymo research, Catalog#D4013). Quantitative PCR was performed as previously described. Primer sequences for ChIP-qPCR and antibody information are provided in Supplementary Table 1.

ChIP for RNA-polymerase II and E2F1 was performed as previously described [24]. The immunoprecipitated DNA fragments were quantified by qPCR (see Additional File 1 for primer sequences and antibodies for ChIP). For Pol2-P5 (Phospho-serine 5) immunoprecipitation, 5 × 10^6^ cells were transfected with siRNA against TAZ-AS202 #1 or control oligos and cells were harvested 72 h after transfection. For E2F1 immunoprecipitation, 20 × 10^6^ cells were used. 2 μg of each antibody were used. For each experiment, a chromatin amount corresponding to 1% of chromatin used for immunoprecipitation was kept as input control.

### Actinomycin D, Cycloheximide and MG-132 treatments

For Actinomycin D treatment, 12 h after siRNA transfection, cells were treated for 8 h with actinomycin D (Sigma-Aldrich, Milan, Italy) at the concentration of 5 μg/ml or DMSO (MOCK). For cycloheximide treatment, 6 h after transfection, cells were treated for 24 h with cycloheximide at the concentration of 50 μg/ml. For MG-132 treatment, 6 h after transfection, cells were treated for 24 h with MG-132 at the concentration of 10 μM.

### Patient selection and RNA-scope

Formalin-fixed, paraffin-embedded (FFPE) from lung cancer patients were retrieved from the Biobank of AUSL-IRCCS di Reggio Emilia. This study was authorized by local Ethical Committee (Comitato Etico dell’Area Vasta Emilia Nord, authorization number 870/2020/TESS/IRCCSRE) and conducted according to Helsinki declaration. Written informed consensus was obtained from all patients. For RNA-scope experiment, nine patients were selected. For patient’s details, see Supplementary Table 2. RNA-scope experiment was performed on FFPE samples of selected patients following manufacture protocol from ADC-bio (https://acdbio.com/rnascope%E2%84%A2-basescope%E2%84%A2-and-mirnascope%E2%84%A2assays). At least four fields for each well were captured using a Nikon microscope. To obtain the graph, we normalized the number of red dots with the number of cells in each field and we reported in graph as percentage (red dots/number of cells) *100.

### Statistical analysis

Statistical analyses were performed using GraphPad Prism Software (GraphPad, San Diego, CA, USA). Statistical significance was determined using the Student *t* test. The variance between the groups that have been compared was assumed to be similar. Each experiment was replicated two to five times.

## Results

### lncRNA TAZ-AS202 has a TAZ-independent pro-oncogenic role in NSCLC

**Fig. 1:**
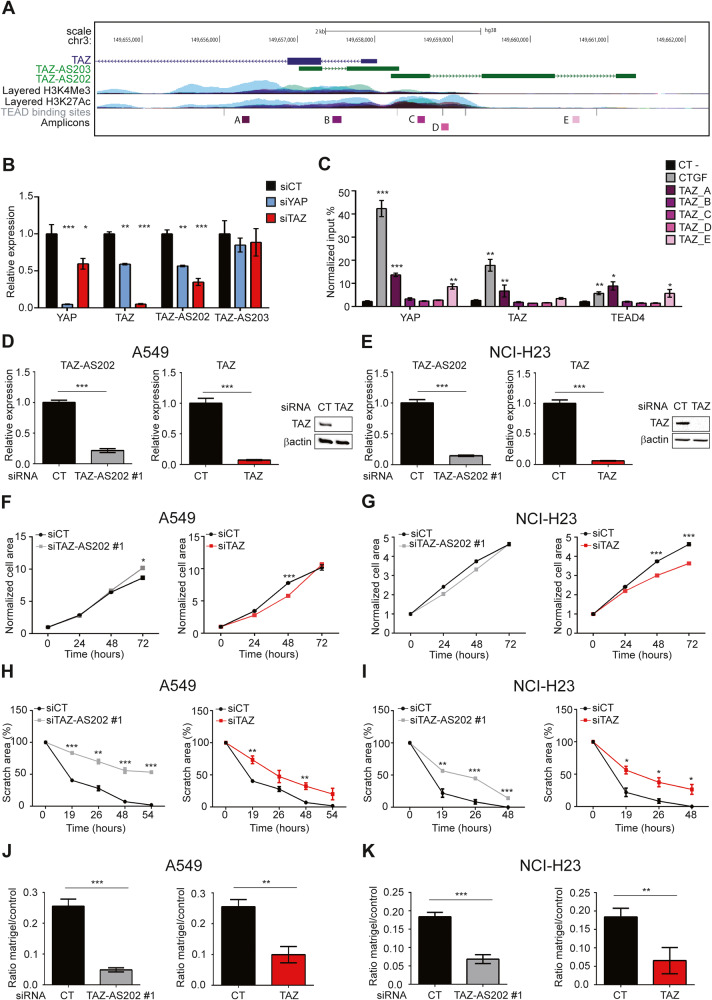
Characterization of TAZ-AS202 as a pro-oncogenic factor in NSCLC. **A** Schematic representation of the TAZ genomic locus on chromosome 3, showing the TAZ transcriptional starting site and the antisense TAZ-AS202 and TAZ-AS203 transcripts. The layered H3K4Me3 and H3K27Ac tracks mark the TAZ promoter. **B** YAP, TAZ, TAZ-AS202 or TAZ-AS203 expression, measured by qRT-PCR in A549 cells transfected with control siRNA (siCT) or siRNA against YAP (siYAP) or TAZ (siTAZ). Data are expressed as mean ± SEM; *p < 0.05; **p < 0.01; ***p < 0.001; *N* = 3. **C** Chromatin Immunoprecipitation (ChIP) analysis of the enrichment of YAP, TAZ or TEAD4 on the TAZ promoter locus. CTGF promoter was used as a positive control. The negative control region (CT-) is an intergenic region on chromosome 19. Enrichments are expressed as input percentage and normalized on IgG signal for each amplicon. Data are expressed as mean ± SEM; *p < 0.05; **p < 0.01; ***p < 0.001; *N* = 3. TAZ-AS202 or TAZ expression, measured by qRT-PCR or Western blot (insets) in A549 (**D**) or NCI-H23 (**E**) NSCLC cell lines upon transfection with control siRNA (CT) or siRNA against TAZ or TAZ-AS202 (siRNA #1). The β-actin is used as a loading control of the Western blot. Data are expressed as mean ± SEM; ***p < 0.001; *N* = 3. Proliferation curves of A549 (**F**) or NCI-H23 (**G**) cells transfected with control siRNA (siCT) or siRNA against TAZ (siTAZ) or TAZ-AS202 (siTAZ-AS202 #1). Cell area has been normalized on time 0. Data are expressed as mean ± SEM; *p < 0.05; ***p < 0.001; *N* = 3. Scratch wound-healing assay in A549 (**H**) or NCI-H23 cells (**I**) transfected with control siRNA (siCT) or siRNA against TAZ (siTAZ) or TAZ-AS202 (siTAZ-AS202 #1). Scratch area at each time point is expressed as percentage of scratch area at time 0. Data are expressed as mean ± SEM; *p < 0.05; **p < 0.01; ***p < 0.001; *N* = 3. Invasion assay of A549 (**J**) or NCI-H23 (**K**) cells transfected with control siRNA (CT) or siRNA against TAZ or TAZ-AS202 (siRNA #1). The number of invading cells in matrigel inserts has been normalized on invading cells in control inserts. Data are expressed as mean ± SEM; **p < 0.01; ***p < 0.001; *N* = 3.

We identified two lncRNAs transcribed from the TAZ gene locus in antisense orientation relative to the TAZ mRNA. The TAZ-AS202 transcript starts from the TAZ promoter and has no overlap with the TAZ transcript, whereas TAZ-AS203 starts from TAZ exon 2 and partially overlaps with the TAZ transcript (Fig. 1A). This genomic locus contains several TEAD binding sites, suggesting the possibility of an autoregulatory loop of TAZ on its own promoter. siRNA-mediated downregulation of YAP or TAZ expression negatively affects the expression of TAZ and TAZ-AS202, whereas it has no effect on the expression of TAZ-AS203 (Fig. 1B). We also confirmed by ChIP that YAP, TAZ and TEAD4 can directly bind this region (Fig. 1C).

**Fig. 2:**
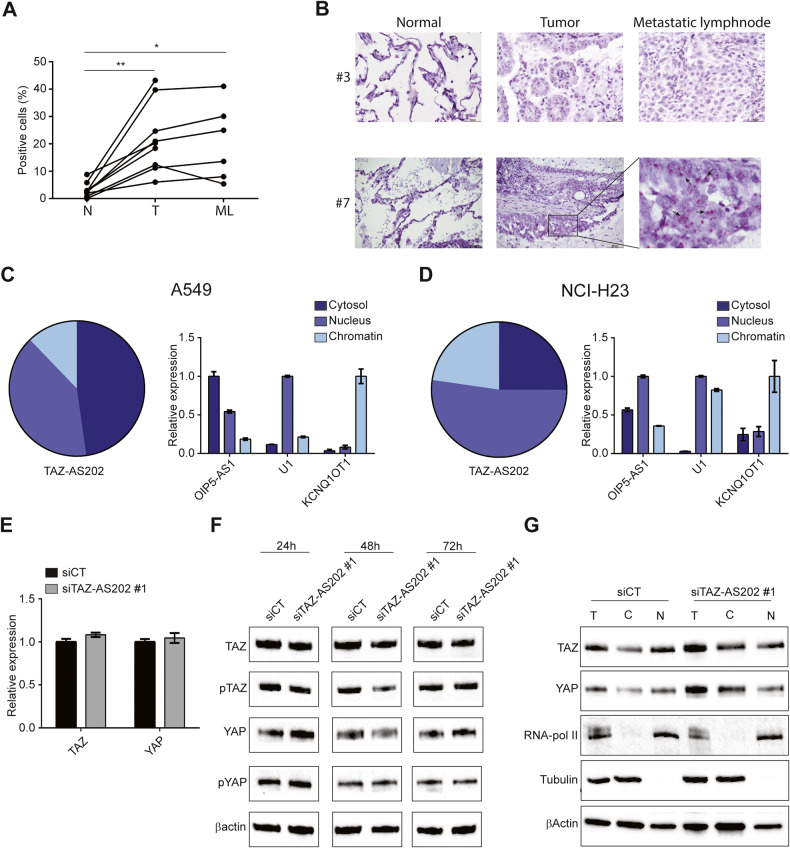
TAZ-AS202 is equally distributed between nucleus and cytosol and acts independently on TAZ or YAP. **A** TAZ-AS202 expression in a set of surgical samples from our Institute Biobank, including normal lung tissues (N; *N* = 9), corresponding adenocarcinoma tissues (T; *N* = 9) and metastatic lymphnodes (ML; *N* = 6). The samples were stained with a RNA-scope probe against TAZ-AS202 and the percentage of positive cells was manually counted in at least three fields per sample. *p < 0.05; **p < 0.01. **B** Representative images of RNA-scope analysis on two patients. Slides were counter-stained with hematoxylin. Arrows indicate some of the red dots, representing TAZ-AS202 signals. Subcellular distribution of TAZ-AS202 in A549 (**C**) or NCI-H23 (**D**) cell lines. Typical cytosolic (OIP5-AS1), nuclear (U1) or chromatin-associated (KCNQ1OT1) RNAs have been analyzed in the same extracts and are shown as control. **E** Relative TAZ or YAP expression, measured by qRT-PCR in A549 cells transfected with control siRNA (siCT) or siRNA against TAZ-AS202 (siTAZ-AS202 #1). Data are expressed as mean ± SEM; *N* = 3. **F** Western blot showing TAZ, phospho-TAZ (pTAZ), YAP or phospho-YAP (pYAP) expression at the indicated time points after transfection with control siRNA (siCT) or siRNA against TAZ-AS202 (siTAZ-AS202 #1). The β-actin is used as a loading control. **G** Analysis of TAZ and YAP sub-cellular localization upon transfection with control siRNA (siCT) or siRNA against TAZ-AS202 (siTAZ-AS202 #1). T indicates total extract, C indicates cytosolic extract and N indicates nuclear extract. RNA-pol II and Tubulin have been analyzed as fractionation controls, representing a nuclear and a cytosolic protein, respectively. The β-actin is used as a loading control.

To investigate the biological role of these two lncRNAs, we silenced their expression in two NSCLC models, the A549 and the NCI-H23 cell lines (Fig. 1D, E and Supplementary Figure 1A–D). The knockdown of TAZ induced a slight proliferation impairment in both cell lines, whereas the knockdown of TAZ-AS202 did not significantly affected this feature (Fig. 1F, G and Supplementary Fig. 1E, F). Importantly, the silencing of TAZ-AS202 had a negative effect on cell migration and invasion in both cell lines, comparable to that observed with TAZ silencing (Fig. 1H–K and Supplementary Fig. 1G–N). On the contrary, TAZ-AS203 silencing did not significantly alter any cell lines feature (Supplementary Fig. 1O–R). These results suggest that TAZ-AS202 supports aggressiveness of NSCLC, while TAZ-AS203 does not have the same role in NSCLC biology. Based on these results, we focused our experiments only on TAZ-AS202. First, we analyzed the TAZ-AS202 expression by RNA-scope in a set of NSCLC patients’ tissues retrieved from our Institute Biobank. TAZ-AS202 is overexpressed in all NSCLC tissues and lymph node metastases, as compared with surrounding healthy lung tissues (Fig. 2A, B). This result further supports a pro-oncogenic role of TAZ-AS202 in NSCLC.

The biological function of lncRNAs is often linked to their sub-cellular localization. To start investigating TAZ-AS202 function, we analyzed TAZ-AS202 expression in sub-cellular compartments of both A549 and NCI-H23 cell lines. This experiment showed that TAZ-AS202 is equally distributed between nucleus and cytosol in A549 cells, whereas it is mainly nuclear in NCI-H23 cells, with a small chromatin-associated fraction in both cell lines (Fig. 2C, D and Supplementary Fig. 2A, B). These results suggest that TAZ-AS202 may have both nuclear and cytoplasmic functions or may shuttle between the two cellular compartments. Due to its partial nuclear/chromatin localization, we first hypothesized that TAZ-AS202 may be implicated in TAZ transcriptional regulation. Surprisingly, TAZ-AS202 silencing did not affect TAZ expression neither at mRNA nor at protein level (Fig. 2E, F). Moreover, TAZ-AS202 silencing did not affect TAZ phosphorylation and sub-cellular localization (Fig. 2F, G). As a control, we also analyzed YAP, the TAZ paralogue, which is transcribed from a different chromosome, and did not observe any change in its expression, phosphorylation or sub-cellular localization upon TAZ-AS202 silencing (Fig. 2E–G). Taken together, these results indicate that TAZ-AS202 exerts pro-oncogenic functions in NSCLC through a YAP/TAZ independent mechanism.

### TAZ-AS202 promotes pro-oncogenic features by supporting the expression of pro-oncogenic pathways, including WNT and EPH-Ephrin

**Fig. 3:**
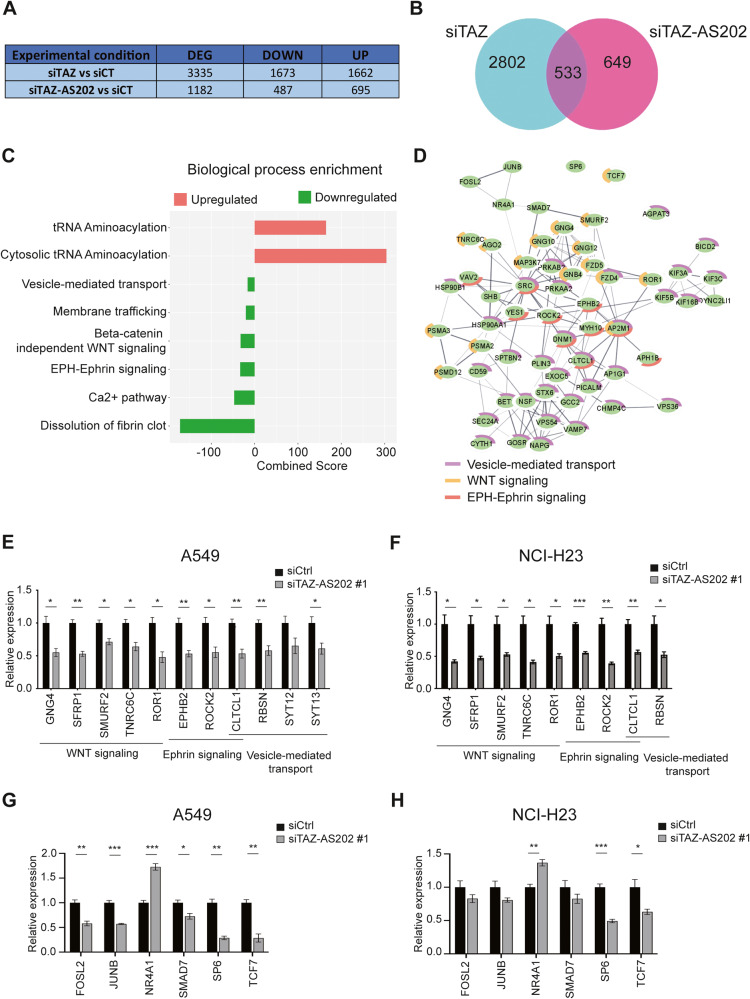
TAZ-AS202 regulates a set of genes belonging to WNT and EPH-Ephrin signaling. **A** Table showing the number of significantly deregulated genes in A549 transfected with siRNA against TAZ (siTAZ) or siRNA against TAZ-AS202 (siTAZ-AS202 #1), in comparison with cells transfected with control siRNA (siCT). **B** Venn diagram showing the overlap between genes deregulated by TAZ siRNA and genes deregulated by TAZ-AS202 siRNA. **C** Reactome enrichment analysis of the pathways deregulated in cells transfected with TAZ-AS202 siRNA compared with control siRNA. **D** String diagram showing connections among the downregulated genes belonging to the indicated pathways. Image modified with Cytoscape. Validation by qRT-PCR of a set of genes deregulated upon transfection with siRNA against TAZ-AS202 (siTAZ-AS202 #1) relative to control siRNA (siCtrl) in A549 (**E**) and NCI-H23 (**F**) cells. The corresponding pathways are indicated below the gene names. Data are expressed as mean ± SEM; *p < 0.05; **p < 0.01; ***p < 0.001; *N* = 3. Validation by qRT-PCR of a set of transcription factors deregulated upon transfection with siRNA against TAZ-AS202 (siTAZ-AS202 #1) relative to control siRNA (siCtrl) in A549 (**G**) and NCI-H23 (**H**) cells. Data are expressed as mean ± SEM; *p < 0.05; **p < 0.01; ***p < 0.001; *N* = 3.

To explore the molecular events underlying TAZ-AS202 pro-oncogenic function, we compared, by RNA-seq, the transcriptome of A549 control cells with that of cells silenced for TAZ-AS202. In parallel, we also sequenced the RNA from cells silenced for TAZ. 1182 differentially expressed genes were found for TAZ-AS202 silencing and 3335 for TAZ. Genes were equally distributed between upregulated and downregulated (Fig. 3A). The samples derived from cells silenced for TAZ-AS202 or TAZ clustered separately at principal component analysis (Supplementary Fig. 2C) and the number of overlapping deregulated genes was 533, less than 50% of TAZ-AS202 target genes and about 16% of TAZ target genes (Fig. 3B). These results further support a TAZ-independent mechanism of action of TAZ-AS202. Next, we performed a biological process enrichment analysis of siTAZ-AS202 differentially expressed genes and found that downregulated genes are significantly enriched in pathways involved in cell-cell interaction and cancer progression, such as EPH-Ephrin signaling, WNT pathway and vesicle-mediated transport (Fig. 3C, D). Since the deregulation of these pathways may explain the reduced aggressive potential that we observed, we proceeded to validate a set of genes belonging to these pathways in both A549 and NCI-H23 cells (Fig. 3E, F).

Interestingly, these genes are also functionally inter-connected with each other and include a set of 8 transcription factor genes that have been previously implicated in cancer and could be responsible for coordinated deregulation (Fig. 3D). qRT-PCR analysis confirmed the downregulation upon TAZ-AS202 silencing for 5 of the 8 transcription factor genes: FOSL2, JUNB, TCF7, SMAD7 and SP6, and also confirmed the upregulation of NR4A1 (Fig. 3G, H).

**Fig. 4:**
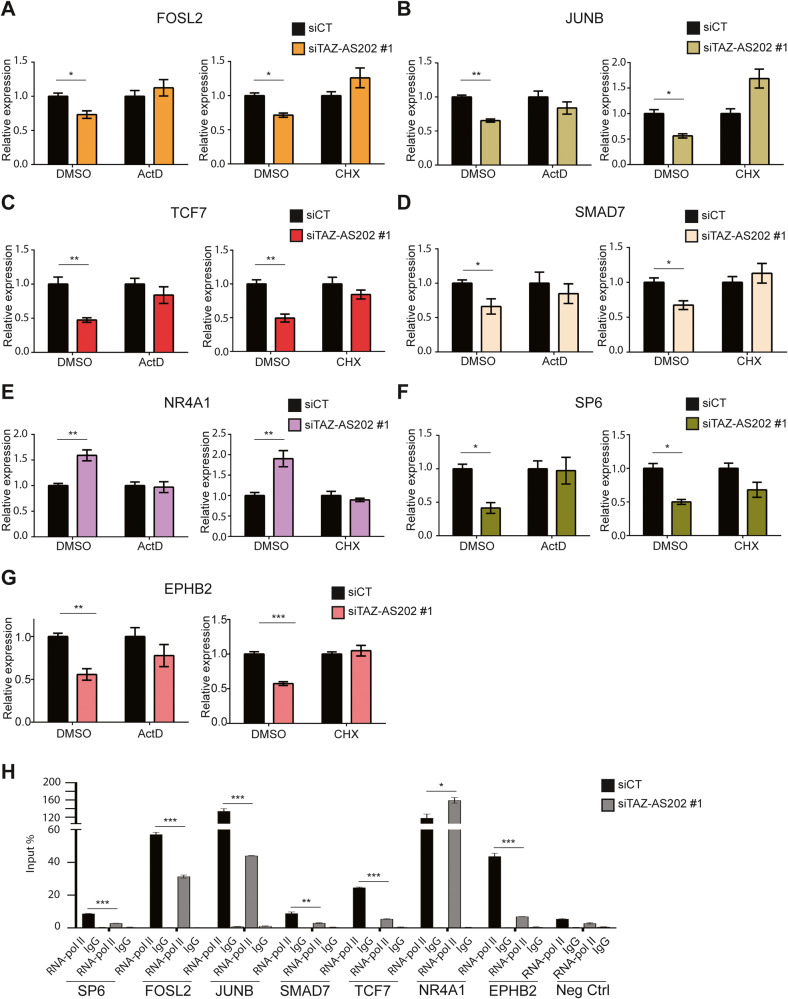
TAZ-AS202 deregulates its target genes through an indirect transcriptional mechanism. **A**–**G** Expression analysis of the indicated genes, measured by qRT-PCR, in A549 cells transfected with control siRNA (siCT) or siRNA against TAZ-AS202 (siTAZ-AS202 #1) and treated with actinomycin D (ActD), cycloheximide (CHX) or solvent dimethysulphoxide (DMSO) as a control. Data are expressed as mean ± SEM; *p < 0.05; **p < 0.01; *N* = 3. **H** Chromatin immunoprecipitation (ChIP) analysis of RNA-polymerase II (RNA-pol II) enrichment on the promoters of the indicated genes in A549 cells transfected with control siRNA (siCT) or siRNA against TAZ-AS202 (siTAZ-AS202 #1). Normal IgG are used as negative control. The negative control region (Neg Ctrl) is an intergenic region on chromosome 6. Enrichment is expressed as input percentage. Data are expressed as mean ± SEM; ***p < 0.001; *N* = 3.

To dissect the molecular mechanism through which TAZ-AS202 regulates the expression of its target genes, we silenced TAZ-AS202 in presence of actinomycin D (ActD) or cycloheximide (CHX), inhibitors of transcription and protein synthesis, respectively. As shown in Fig. 4A–G, both ActD and CHX abolished the effect of TAZ-AS202 silencing on all the target genes, including both transcription factors and putative effector genes, like EPHB2. These results indicate that active transcription and translation are required for TAZ-AS202 function, pointing to a transcriptional indirect mechanism. To confirm this notion, we performed a ChIP experiment, demonstrating that the silencing of TAZ-AS202 reduced RNA polymerase II (RNA-pol II) occupancy of the promoter of target genes which are downregulated, whereas it has the opposite effect on the promoter of NR4A1, which is upregulated (Fig. 4H).

### TAZ-AS202 regulates its targets expression through the regulation of the E2F1 transcription factor protein level

**Fig. 5:**
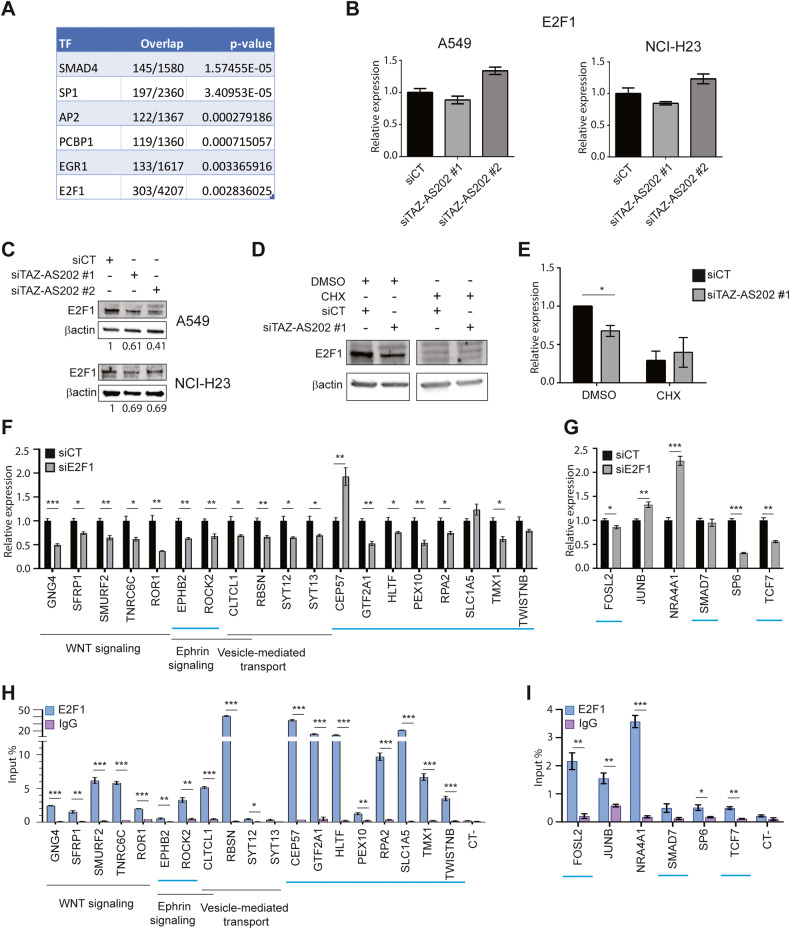
TAZ-AS202 regulates its target genes through increase of E2F1 protein. **A** Enrichment analysis of transcription factors binding sites on the promoter of genes deregulated in cells transfected with TAZ-AS202 siRNA compared with control siRNA. For each significantly enriched transcription factor (TF), the number of overlapping genes and *p*-values are shown. **B** E2F1 expression in A549 or NCI-H23 cells transfected with control siRNA (siCT) or two different siRNA against TAZ-AS202 (siTAZ-AS202 #1 and siTAZ-AS202 #2), measured by qRT-PCR. Data are expressed as mean ± SEM; *N* = 3. **C** E2F1 protein expression, measured by Western blot analysis, in A549 or NCI-H23 cells transfected with control siRNA (siCT) or two different siRNA against TAZ-AS202 (siTAZ-AS202 #1 and siTAZ-AS202 #2). E2F1 bands have been quantified and normalized on β-actin. Normalized values are shown below the blots. **D** Western blot analysis of E2F1 protein expression in A549 cells transfected with control siRNA (siCT) or siRNA against TAZ-AS202 (siTAZ-AS202 #1) and treated cycloheximide (CHX) or solvent dimethyl-sulphoxide (DMSO). The β-actin is used as a loading control. **E** Quantification of E2F1 protein in two independent Western blot experiments performed on A549 cells transfected with control siRNA (siCT) or siRNA against TAZ-AS202 (siTAZ-AS202 #1) and treated with cycloheximide (CHX) or solvent dimethyl-sulphoxide (DMSO). E2F1 signals have been normalized on β-actin and expressed as relative to siCT DMSO sample. **F** Expression of a set of TAZ-AS202 target genes in A549 cells transfected with control siRNA (siCT) or siRNA against E2F1 (siE2F1), measured by qRT-PCR. The corresponding pathways are indicated below the gene names. The light blue line indicates the genes predicted to carry E2F1 binding sites in the promoter. Data are expressed as mean ± SEM; *p < 0.05; **p < 0.01; ***p < 0.001; *N* = 3. **G** Expression of a set of transcription factors in A549 cells transfected with control siRNA (siCT) or siRNA against E2F1 (siE2F1), measured by qRT-PCR. The light blue line indicates the genes predicted to carry E2F1 binding sites in the promoter. Data are expressed as mean ± SEM; *p < 0.05; **p < 0.01; ***p < 0.001; *N* = 3. **H**, **I** Chromatin immunoprecipitation (ChIP) analysis of E2F1 enrichment on the promoters of the genes analyzed in **F**, **G**. Normal rabbit IgG are used as negative control. The negative control region (CT-) is an intergenic region on chromosome 6. Enrichment is expressed as input percentage. Data are expressed as mean ± SEM; *p < 0.05; **p < 0.01; ***p < 0.001; *N* = 3.

Since all the identified targets of TAZ-AS202 are deregulated through an indirect mechanism, we reasoned that TAZ-AS202 may influence at post-transcriptional level one or more transcription factors, that in turn would be responsible for the deregulation of the other genes. To identify this putative mediator(s) of TAZ-AS202 activity, we searched the promoter of genes deregulated by TAZ-AS202 silencing for transcription factors binding sites. From this analysis, six transcription factors binding sites resulted significantly enriched: SMAD4, SP1, AP2, PCBP1, EGR1 and E2F1 (Fig. 5A). We verified whether these transcription factors were deregulated in cells silenced for TAZ-AS202 and we found that E2F1 was downregulated at protein level but not at mRNA level (Fig. 5B, C). The other transcription factors were not affected by TAZ-AS202 silencing (Supplementary Fig. 3A–E). These results confirmed our RNA-seq data, that showed no deregulation of any of these transcription factors at mRNA level and fit with our hypothesis that a post-transcriptional mechanism mediates TAZ-AS202 activity.

Notably, a recent paper reported that the cytosolic lncRNA lncNB1 regulates E2F1 protein level by increasing its translation rate [25]. Thus, to verify whether TAZ-AS202 regulates E2F1 through a similar mechanism, we repeated the silencing experiment in presence of translation inhibitor CHX. Strikingly, E2F1 protein downregulation by TAZ-AS202 was inhibited by CHX treatment (Fig. 5D, E), suggesting that TAZ-AS202 may regulate E2F1 by supporting its translation. E2F1 may in turn transcriptionally activate the expression of the TAZ-AS202 target genes. In accordance with this hypothesis, the silencing of E2F1 produced a deregulation of all the previously validated TAZ-AS202 target genes with a similar trend (Figs. 3E, F and 5F). In addition, we also selected a further set of genes deregulated by TAZ-AS202 from the RNA-seq analysis and predicted to carry more than one E2F binding site on their promoters. Notably, most of these genes were deregulated by E2F1 silencing as well (Fig. 5F). Furthermore, E2F1 silencing deregulated also the expression of 5 out of 6 transcription factors identified and validated as TAZ-AS202 indirect targets from the RNA-seq analysis (Figs. 3G, H and 5G). Intriguingly, only a fraction of the genes selected for the first RNA-seq validation and deregulated by E2F1 were predicted to carry E2F1 binding sites on their promoters. Thus, to verify if E2F1 directly binds to the promoters of these genes, we performed a ChIP experiment with anti-E2F1 antibodies. Strikingly, E2F1 bound the large majority of the promoters, indicating that this transcription factor directly regulated the expression of these genes (Fig. 5H, I).

### E2F1 and EPHB2 are mediators of the TAZ-AS202 pro-oncogenic function

**Fig. 6:**
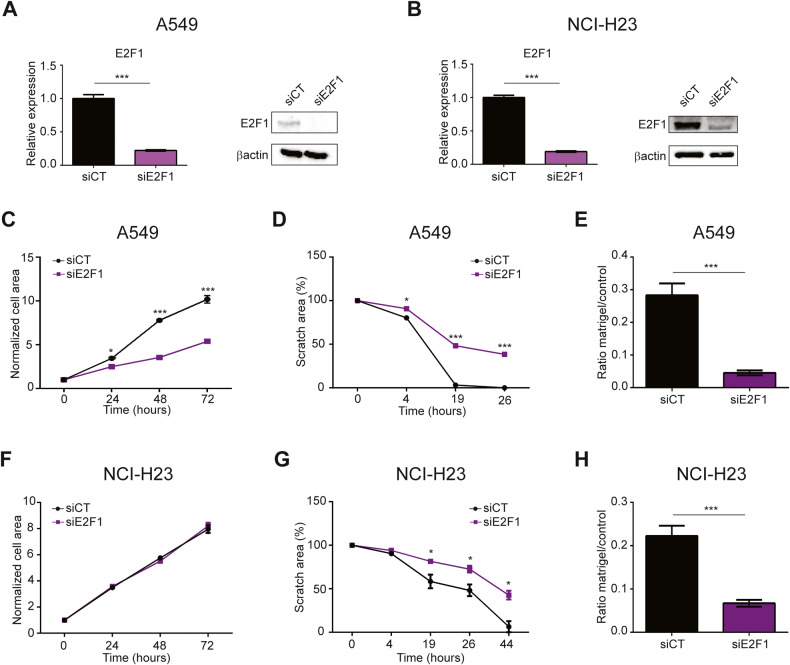
E2F1 promotes NSCLC pro-oncogenic features. E2F1 expression, measured by qRT-PCR or Western blot (insets) in A549 (**A**) or NCI-H23 (**B**) NSCLC cell lines upon transfection with control siRNA (Ctrl) or siRNA against E2F1. The β-actin is used as a loading control of the Western blot. Data are expressed as mean ± SEM; **p < 0.01; ***p < 0.001; *N* = 3. Proliferation curves of A549 (**C**) or NCI-H23 (**F**) cells transfected with control siRNA (siCT) or siRNA against E2F1 (siE2F1). Cell area has been normalized on time 0. Data are expressed as mean ± SEM; *p < 0.05; ***p < 0.001; *N* = 3. Scratch wound-healing assay in A549 (**D**) or NCI-H23 cells (**G**) transfected with control siRNA (siCT) or siRNA against E2F1 (siE2F1). Scratch area at each time point is expressed as percentage of scratch area at time 0. Data are expressed as mean ± SEM; *p < 0.05; **p < 0.01; ***p < 0.001; *N* = 3. Invasion assay of A549 (**E**) or NCI-H23 (**H**) cells transfected with control siRNA (CT) or siRNA against E2F1. The number of invading cells in matrigel inserts has been normalized on invading cells in control inserts. Data are expressed as mean ± SEM; ***p < 0.001; *N* = 3.

To confirm that E2F1 mediated the effect of TAZ-AS202 on pro-oncogenic properties of NSCLC cell lines, we compared cell pro-oncogenic features in control and E2F1 silenced NSCLC cells (Fig. 6A, B). E2F1 silenced cells showed reduced migration and invasion of both A549 and NCI-H23 cell lines, similarly to what we observed in TAZ-AS202 silenced cells (Fig. 6C–H and Supplementary Fig. 3F–I). Strikingly, E2F1 silencing decreased also proliferation in A549 cells but not in NCI-H23, indicating a differential requirement for E2F1 activity in the two cell lines. These data indicates that TAZ-AS202 promotes NSCLC pro-oncogenic properties through regulation of E2F1 protein, which in turn directly regulates the expression of a large set of target genes involved in cancer progression and aggressiveness. We focalized our attention on EPH-Ephrin signaling target genes, since this pathway is known to be involved in cell-cell adhesion, cell shape, cell motility and interaction with the extra-cellular matrix, features that may explain the decreased invasive/migratory phenotype we observed in TAZ-AS202 silenced cells [26]. Notably, among the TAZ-AS202/E2F1 targets we found the EPHB2 receptor, a key protein in this pathway that is overexpressed in lung cancer cells [27].

**Fig. 7:**
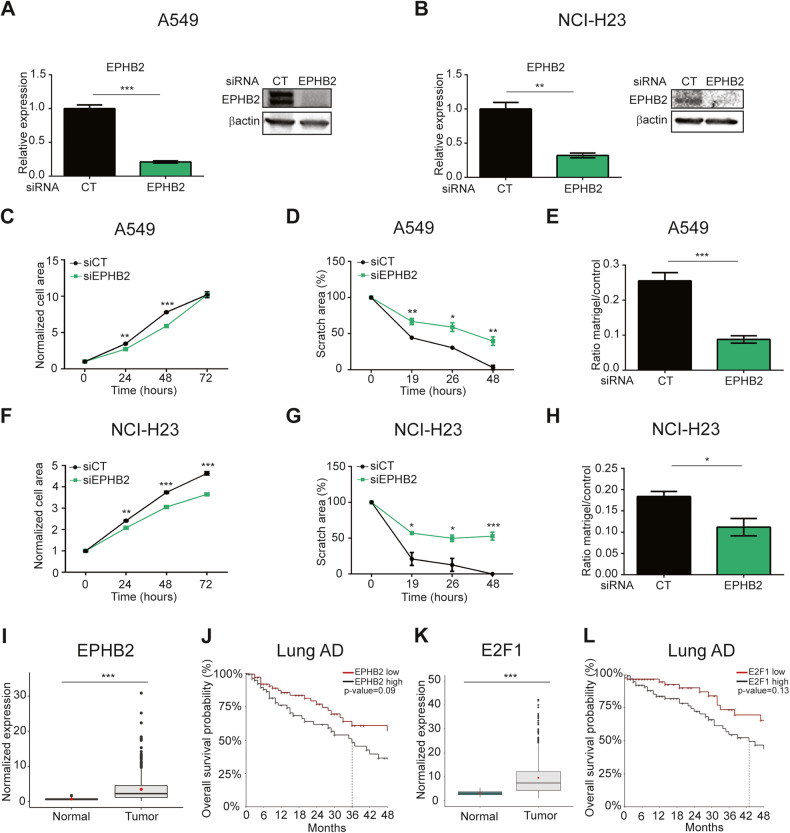
EPHB2 promotes NSCLC pro-oncogenic features. EPHB2 expression, measured by qRT-PCR or Western blot (insets) in A549 (**A**) or NCI-H23 (**B**) NSCLC cell lines upon transfection with control siRNA (CT) or siRNA against EPHB2. The β-actin is used as a loading control of the Western blot. Data are expressed as mean ± SEM; **p < 0.01; ***p < 0.001; *N* = 3. Proliferation curves of A549 (**C**) or NCI-H23 (**F**) cells transfected with control siRNA (siCT) or siRNA against EPHB2 (siEPHB2). Cell area has been normalized on time 0. Data are expressed as mean ± SEM; **p < 0.01; ***p < 0.001; *N* = 3. Scratch wound-healing assay in A549 (**D**) or NCI-H23 cells (**G**) transfected with control siRNA (siCT) or siRNA against EPHB2 (siEPHB2). Scratch area at each time point is expressed as percentage of scratch area at time 0. Data are expressed as mean ± SEM; *p < 0.05; **p < 0.01; ***p < 0.001; *N* = 3. Invasion assay of A549 (**E**) or NCI-H23 (**H**) cells transfected with control siRNA (CT) or siRNA against EPHB2. The number of invading cells in matrigel inserts has been normalized on invading cells in control inserts. Data are expressed as mean ± SEM; ***p < 0.001; *N* = 3. **I** EPHB2 expression in TCGA lung adenocarcinoma cohort samples (*N* = 572, normal = 59, tumor = 513), measured by RNA-Seq. ***p < 0.001. **J** Kaplan-Meier curve representing overall survival probability in patients presenting high (*N* = 129) or low (*N* = 129) levels of EPHB2 expression. *p* = 0.09. **K** E2F1 expression in TCGA lung adenocarcinoma cohort samples (*N* = 572, normal = 59, tumor = 513), measured by RNA-Seq. ***p < 0.001. **L** Kaplan-Meier curve representing overall survival probability in patients presenting high (*N* = 129) or low (*N* = 129) levels of E2F1 expression. *p* = 0.13.

To assess whether EPHB2 downregulation may be sufficient for impaired pro-oncogenic features observed in TAZ-AS202 silenced cells, we performed siRNA-mediated downregulation of EPHB2 (Fig. 7A, B). Notably, the silencing of EPHB2 mirrored the phenotype of TAZ-AS202 and E2F1 silenced cells, resulting in migration and invasion marked reduction in both A549 and NCI-H23 cell lines (Fig. 7C–H and Supplementary Fig. 3J–M).

These results suggest that the pro-oncogenic properties of TAZ-AS202 are at least in part mediated by modulation of the protein level of the transcription factor E2F1 that in turn regulates the expression of EPHB2 and possibly other effectors, leading to increased cancer cells aggressiveness.

To confirm the pathological role of this E2F1/EPHB2 axis in lung cancer patients, we analyzed the expression of these genes in the TCGA lung adenocarcinoma cohort. We found that both E2F1 and EPHB2 expression is increased in tumor tissue compared to healthy lung and that high expression is associated with a trend towards worse prognosis, even if not reaching statistical significance (Fig. 7I–L). These results confirmed a pro-oncogenic role of E2F1 and EPHB2 in NSCLC and supported their role in mediating TAZ-AS202 effect on cancer cell properties.

## Discussion

In this work, we identified and characterized the function of a novel lncRNA, TAZ-AS202, which is transcribed from the TAZ gene promoter region in antisense orientation. We showed that this transcript was more abundant in lung cancer cells compared to surrounding healthy lung tissue, suggesting that its expression may function in tumor development and/or progression. Supporting this notion, we observed that silencing TAZ-AS202 attenuated the migratory and invasive potential of NSCLC cell lines.

Because of the TAZ-AS202 genomic position and the partial nuclear/chromatin localization, we first hypothesized that it could have a role in regulating TAZ expression in cis. Surprisingly, we did not observe any change in TAZ or YAP in terms of mRNA or protein level, phosphorylation or subcellular localization. Since we downregulated TAZ-AS202 expression at post-transcriptional level through a siRNA approach, we could not exclude that the TAZ-AS202 transcription itself may influence the transcription of the adjacent TAZ gene. However, since we observed a relevant effect of TAZ-AS202 silencing on cell lines properties, we focused on the molecular mechanism underlying this effect, which is dependent on the TAZ-AS202 transcript and not on its transcription itself.

Conversely, the expression of both TAZ-AS202 and TAZ is under the control of YAP/TAZ/TEAD, as demonstrated by our qRT-PCR and ChIP experiments. This implicates the presence of an auto-regulatory loop, involving the positive regulation of the shared TAZ/TAZ-AS202 promoter by YAP/TAZ. Moreover, this finding suggests that the YAP/TAZ function in cancer cells may be mediated at least in part by TAZ-AS202.

Based on these observations, we considered that TAZ-AS202 has mainly TAZ-independent functions and we proceeded to characterize these functions. Strikingly, we found a set of genes deregulated by TAZ-AS202 silencing that may explain the effect of this lncRNA on lung cancer cell properties, belonging to different cancer-associated pathways, such as WNT and EPH-Ephrin signaling [28, 29]. After validating a subset of these genes, we concentrated on dissecting the molecular mechanism through which TAZ-AS202 regulates the expression of its target genes.

We showed that TAZ-AS202 increases the level of E2F1 protein, without changing its mRNA levels. E2F1 is a key transcription factor for cell cycle progression, whose levels are tightly regulated during cell cycle [8]. Its levels typically peak at the G1-S phase transition, being its activity required for S phase entry and DNA duplication [30]. E2F1 activity is tightly regulated via multiple mechanisms including transcription, mRNA and protein stability, post-translational modification and interaction with different binding partners [8]. In particular, it has been shown that E2F1 protein translation is regulated by lncRNA lncNB1, through direct binding to the ribosomal subunit RPL35 [25]. In this work, we showed that silencing of TAZ-AS202 decreased E2F1 protein without affecting mRNA levels. The treatment with protein synthesis inhibitor CHX abolished this modulation, indicating that new protein translation is required. This may suggest that TAZ-AS202, similarly to lncNB1, is involved in regulating E2F1 protein translation. In alternative, TAZ-AS202 may regulate E2F1 protein level through an indirect mechanism, involving protein synthesis of a further unknown factor. Additional experiments will be required to dissect this mechanism.

Intriguingly, most of the TAZ-AS202 target genes were also deregulated upon E2F1 silencing, including EPHB2 and a set of transcriptional factors whose expression we previously observed to be indirectly controlled by TAZ-AS202. These results support the role of E2F1 as principal mediator of TAZ-AS202 activity and suggest that the E2F1-dependent deregulation of other transcription factors may participate to define the expression of TAZ-AS202 target genes. In addition, we show that E2F1 occupies the promoter region of virtually all the considered target genes, indicating that it directly regulates their expression. Intriguingly, many of these promoters lack a canonical E2F1 binding site. This finding is in line with a report, showing that only 30% of genes bound by E2F1 carry a canonical binding site in the promoter and supports a mechanism of E2F1 binding on its target genes which is independent on classical DNA binding [31]. However, binding sites for other E2F transcription factors are present on nearly all the promoters (data not shown), suggesting that an alternative explanation may be a scarcely specific recognition of the cognate DNA element.

To confirm that the phenotype we observed in TAZ-AS202 silenced cells was dependent on decreased E2F1 levels, we silenced E2F1 and we obtained a similar impairment of pro-oncogenic cell properties. This result was quite surprising since E2F1 is considered a master regulator of cell cycle, whereas our cells showed a prominent invasion and migration reduction. In particular, the proliferation of NCI-H23 cell line seemed to be not affected by E2F1 silencing, suggesting that in this context E2F1 may not be strictly required for cell cycle progression. On the other hand, our results indicate that E2F1 is also involved in other pro-oncogenic processes, beside cell cycle, such as migration and invasion. This notion is in line with other reports, showing that E2F1 regulates other tumor features, such as metabolism, epithelial-mesenchymal transition, extracellular matrix remodeling and angiogenesis, ultimately promoting invasion and metastasis [10, 12, 13, 32–34].

Notably, a key gene of the EPH-Ephrin signaling, the receptor EPHB2, is one of the most downregulated genes in our RNA-sequencing dataset. Importantly, the silencing of EPHB2 mirrored the effect of TAZ-AS202 and E2F1 silencing on NSCLC features. Although probably other TAZ-AS202/E2F1 target genes contribute to the phenotype, these data support the role of EPHB2 as mediator of TAZ-AS202 activity. EPHB2 is overexpressed in many tumors, including lung, and exerts mainly a tumor-promoting function [26, 27]. In different cancer contexts, it promotes migration, invasion and angiogenesis, while inhibiting cell adhesion [26, 35, 36]. In NSCLC patients, EPHB2 is overexpressed and its high expression is associated with poor overall survival [27]. Our findings are in line with these reports, demonstrating that EPHB2 can influence migration and invasion also in NSCLC, supporting the notion that this receptor has a pro-oncogenic role also in this cancer type. These results are particularly intriguing, since both small molecules and monoclonal antibodies have been developed, targeting various EPH receptors, including EPHB2 [37, 38]. If our results are confirmed by other studies, these molecules may be considered in clinical trials for NSCLC treatment.

**Fig. 8:**
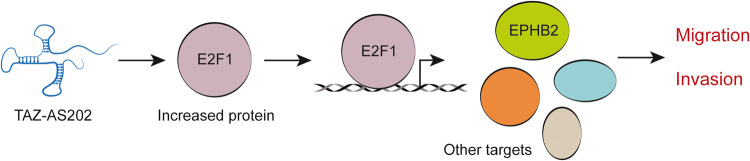
Model of TAZ-AS202 mechanism of action. TAZ-AS202 increases E2F1 protein level, enhancing E2F1 transcriptional activity on a set of cancer-related genes, including EPHB2, which in turn promote cancer cells pro-oncogenic features, such as migration and invasion.

Overall, we characterized the pro-oncogenic function of a novel lncRNA, TAZ-AS202, which supports NSCLC invasion and migration by regulating a set of pro-oncogenic genes, including EPHB2. Mechanistically, TAZ-AS202 increases E2F1 protein, which in turn transcriptionally regulates the TAZ-AS202 target genes (Fig. 8). These results have been obtained in vitro in cell lines of NSCLC, being the lack of functional studies in mice models a limitation of this study. However, our findings unveil a new pro-oncogenic axis, involving TAZ-AS202, E2F1 and EPH-Ephrin signaling, which supports NSCLC tumorigenesis and which can be exploited for future therapeutic targeting.

### Supplementary information

Supplementary Figures

Supplementary Table 1

Supplementary Table 2

Original data files

Reproducibility checklist

## Data Availability

The data underlying this article are available in ArrayExpress at https://www.ebi.ac.uk/biostudies/arrayexpress/studies/, and can be accessed with the accession number E-MTAB-12507.
