# Supplementary material for: The long non-coding RNA TAZ-AS202 promotes lung cancer progression via regulation of the E2F1 transcription factor and activation of Ephrin signaling
Source: Cell Death Dis. 2023 Nov 18;14(11):752. doi: 10.1038/s41419-023-06277-y (PMC10657417; doi:10.1038/s41419-023-06277-y)
Supplement: Supplementary file 1 — Supplementary Figures [file 41419_2023_6277_MOESM1_ESM.pdf]

SUPPLEMENTARY FIGURE 1

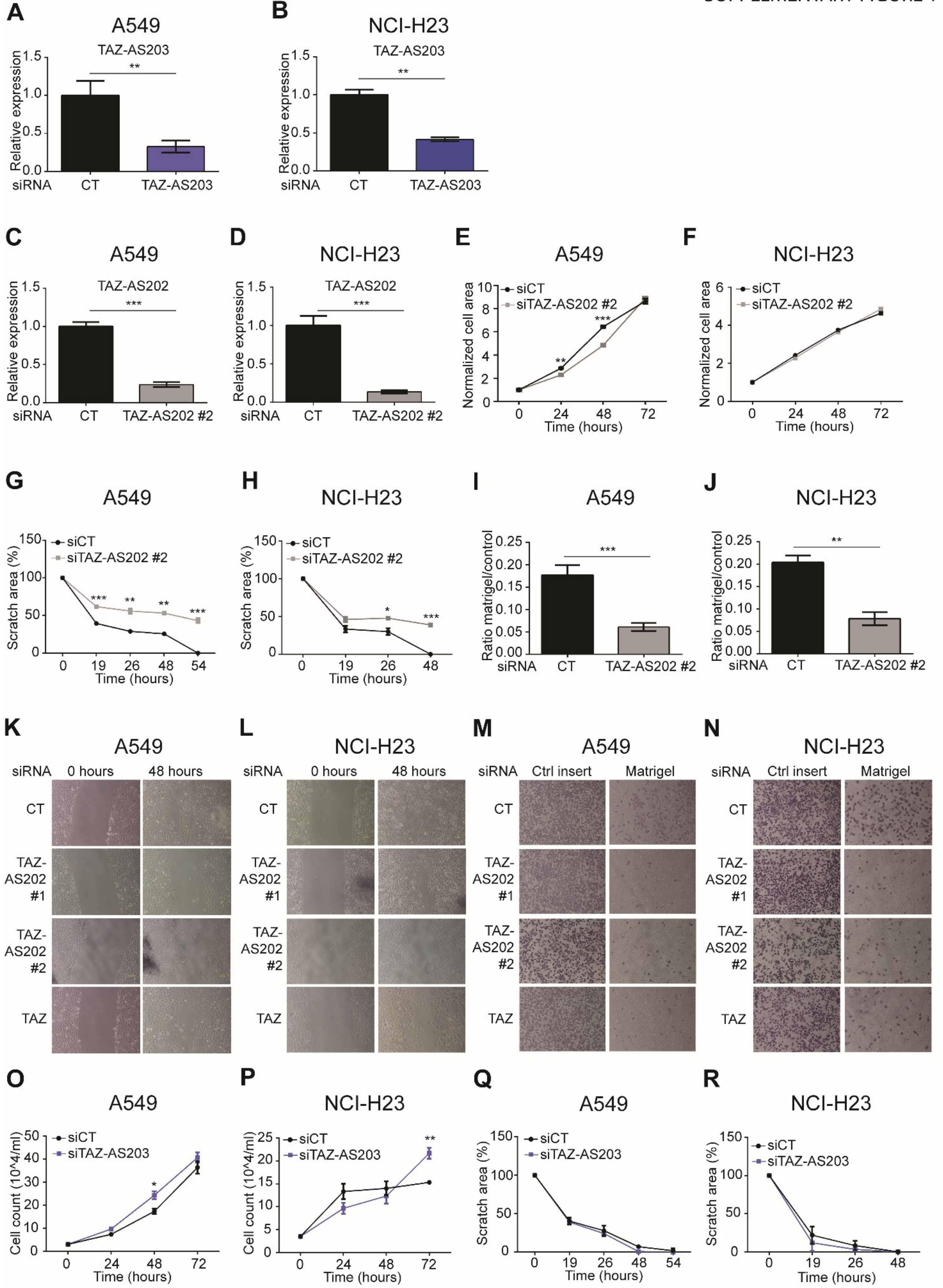

Supplementary Figure 1

A-B) TAZ-AS203 expression, measured by qRT-PCR in A549 (A) or NCI-H23 (B) NSCLC cell lines upon transfection with control siRNA (CT) or siRNA against TAZ-AS203. Data are expressed as mean ±SEM; **=p<0.01; N=3. C-D) TAZ-AS202 expression, measured by qRT-PCR in A549 (C) or NCI-H23 (D) cell lines upon transfection with control siRNA (Ctrl) or siRNA against TAZ-AS202 (siTAZ-AS202 #2). Data are expressed as mean ±SEM; ***=p<0.001; N=3. E-F) Proliferation curves of A549 (E) or NCI-H23 (F) cells transfected with control siRNA (siCT) or siRNA against TAZ-AS202 (siTAZ-AS202 #2). Cell area has been normalized on time 0. Data are expressed as mean ±SEM; **=p<0.01; ***=p<0.001; N=3. G-H) Scratch wound-healing assay in A549 (G) or NCI-H23 cells (H) transfected with control siRNA (siCT) or siRNA against TAZ-AS202 (siTAZ-AS202 #2). Scratch area at each time point is expressed as percentage of scratch area at time 0. Data are expressed as mean ±SEM; *=p<0.05; **=p<0.01; ***=p<0.001; N=3. I-J) Invasion assay of A549 (I) or NCI-H23 (J) cells transfected with control siRNA (CT) or siRNA against TAZ-AS202 (TAZ-AS202 #2). The number of invading cells in matrigel inserts has been normalized on invading cells in control inserts. Data are expressed as mean ±SEM; **=p<0.01; ***=p<0.001; N=3. K-L) Representative images of scratch-wound healing assays in A549 (K) or NCI-H23 (L) cells transfected with control siRNA (CT), with siRNA against TAZ or with two different siRNA against TAZ-AS202. M-N) Representative images of invasion assays in A549 (M) or NCI-H23 (N) cells transfected with control siRNA (CT), with siRNA against TAZ or with two different siRNA against TAZ-AS202. O-P) Proliferation curves of A549 (O) or NCI-H23 (P) cells transfected with control siRNA (siCT) or siRNA against TAZ-AS203. Vital cell counts have been normalized on time 0. Data are expressed as mean ±SEM; *=p<0.05; **=p<0.01; N=3. Q-R) Scratch wound-healing assay in A549 (Q) or NCI-H23 cells (R) transfected with control siRNA (siCT) or siRNA against TAZ-AS203. Scratch area at each time point is expressed as percentage of scratch area at time 0. Data are expressed as mean ±SEM; N=3.

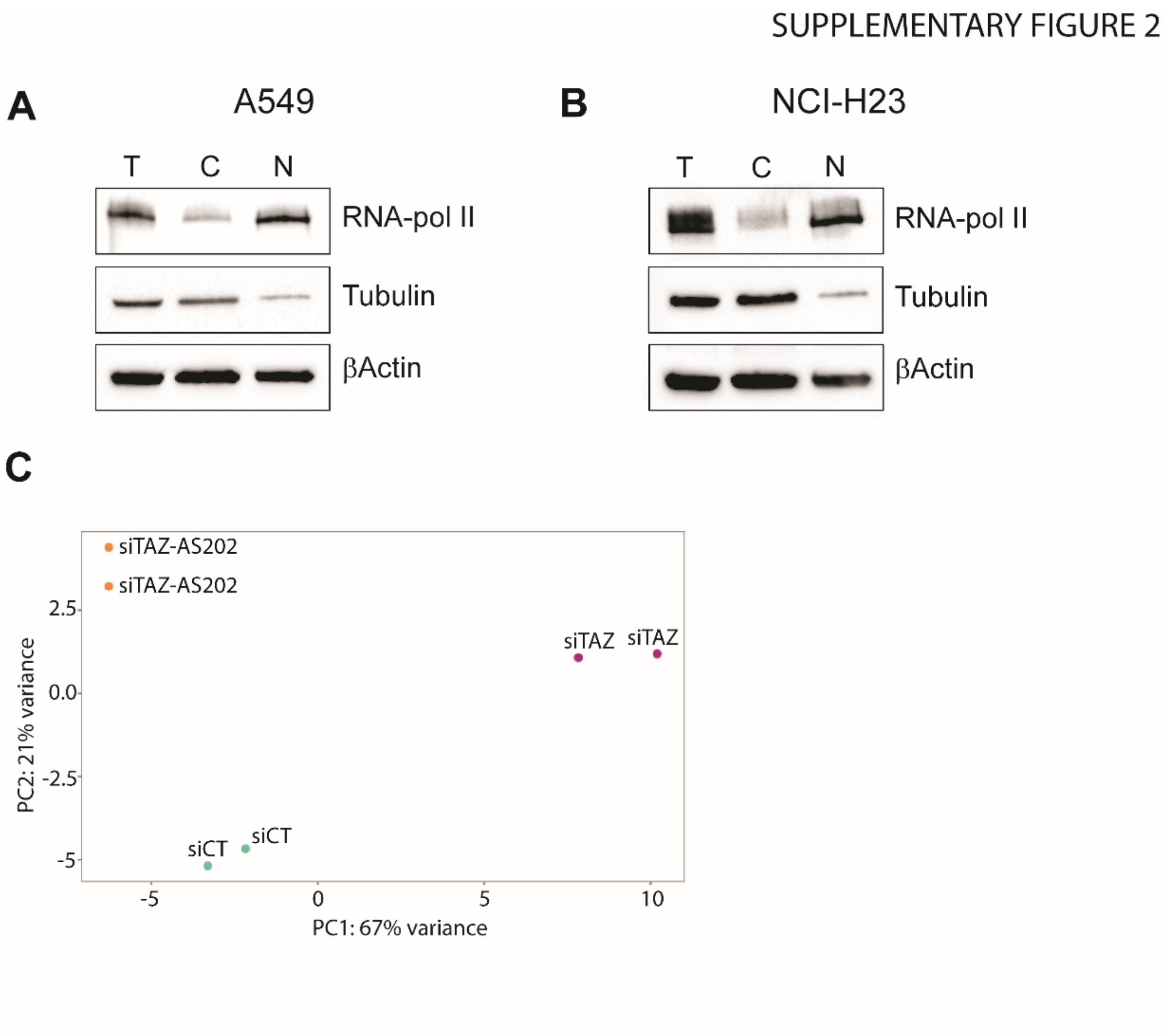

Supplementary Figure 2

A-B) Western blot showing RNA-polymerase II, tubulin and β-actin localization, as a control for cell fractionation experiments in A549 (A) and NCI-H23 (B) cells. T=total extract; C=cytosolic extract; N=nuclear extract. C) Principal component analysis of RNA-sequencing samples derived from A549 cells transfected with control siRNA (siCT), siRNA against TAZ (siTAZ) or siRNA against TAZ-AS202 (siTAZ-AS202).

SUPPLEMENTARY FIGURE 3

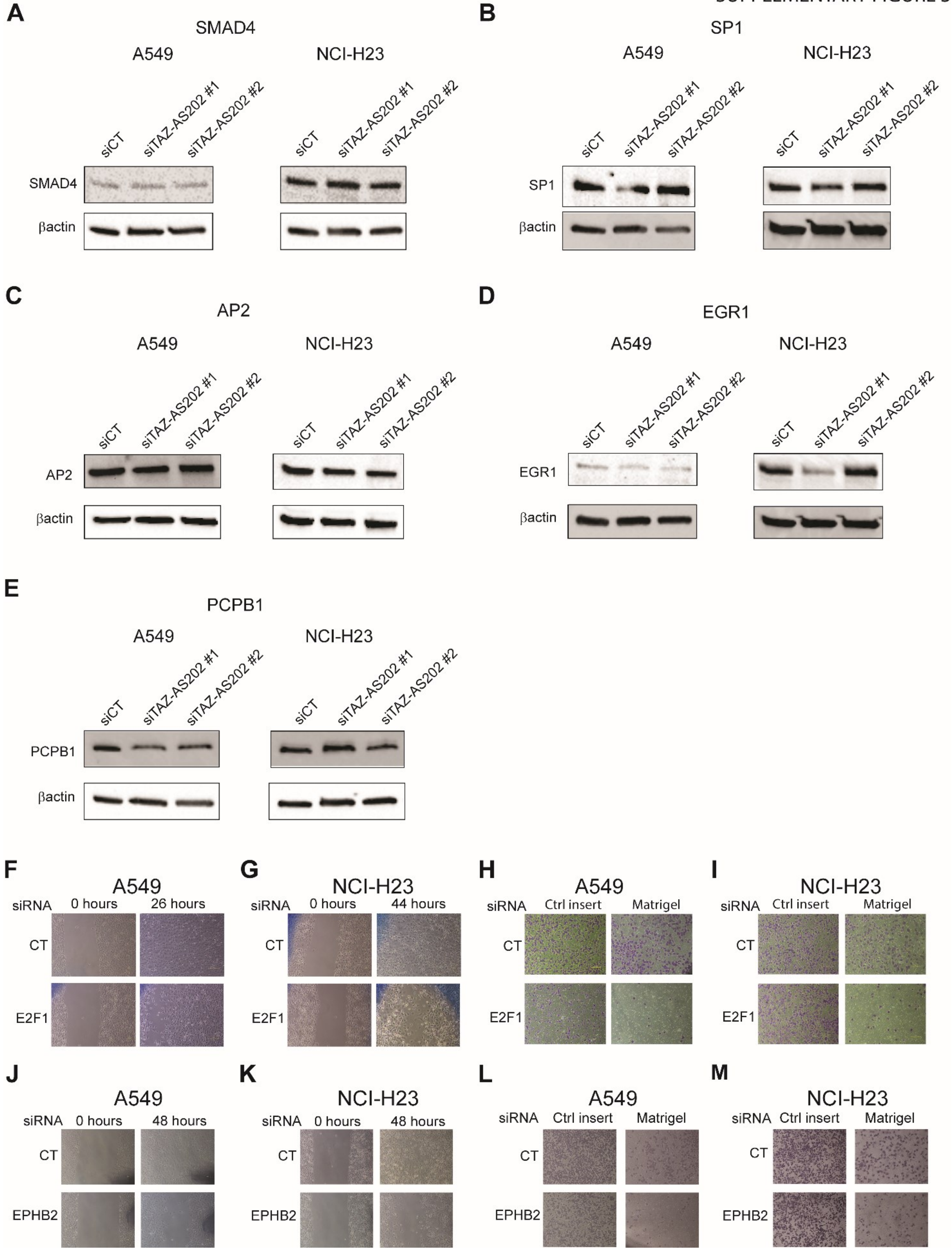

Supplementary Figure 3

A-E) Western blot analysis of the expression of the indicated transcription factors in A549 or NCI-H23 cells transfected with control siRNA (siCT) or two different siRNA against TAZ-AS202 (siTAZ-AS202 #1 and #2). The β-actin is used as a loading control. F-G) Representative images of scratch-wound healing assays in A549 (F) or NCI-H23 (G) cells transfected with control siRNA (CT) or siRNA against E2F1. H-I) Representative images of invasion assays in A549 (H) or NCI-H23 (I) cells transfected with control siRNA (CT) or with siRNA against E2F1. J-K) Representative images of scratch-wound healing assays in A549 (J) or NCI-H23 (K) cells transfected with control siRNA (CT) or siRNA against EPHB2. L-M) Representative images of invasion assays in A549 (L) or NCI-H23 (M) cells transfected with control siRNA (CT) or with siRNA against EPHB2.
