# Supplementary material for: The long non-coding RNA TAZ-AS202 promotes lung cancer progression via regulation of the E2F1 transcription factor and activation of Ephrin signaling
Source: Cell Death Dis. 2023 Nov 18;14(11):752. doi: 10.1038/s41419-023-06277-y (PMC10657417; doi:10.1038/s41419-023-06277-y)

Figure 1D

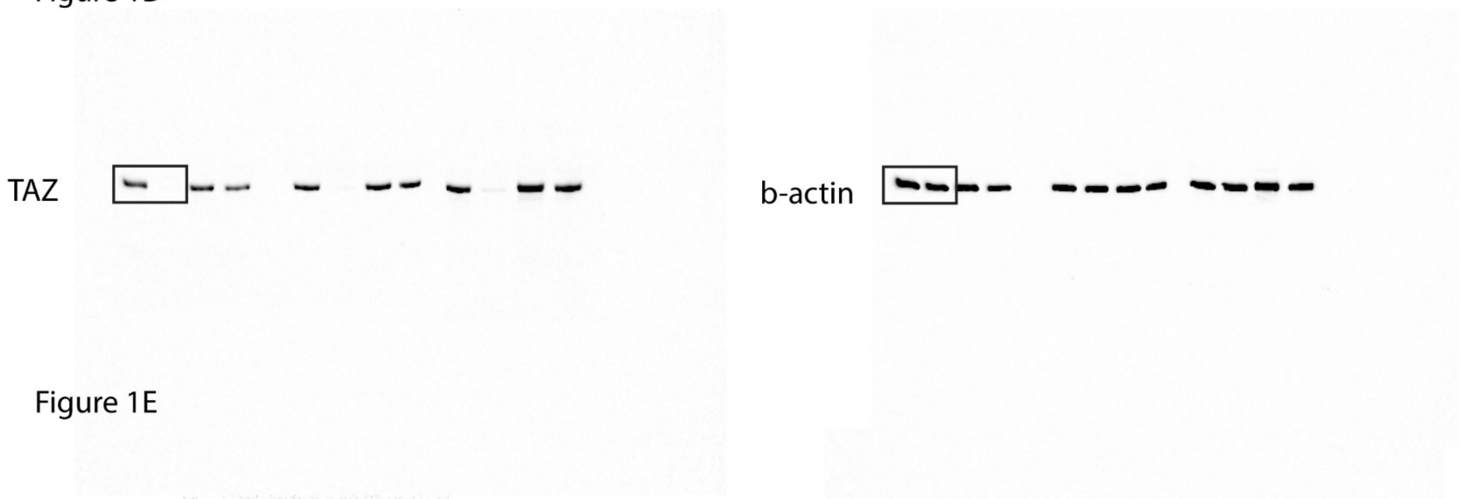

Figure 1E

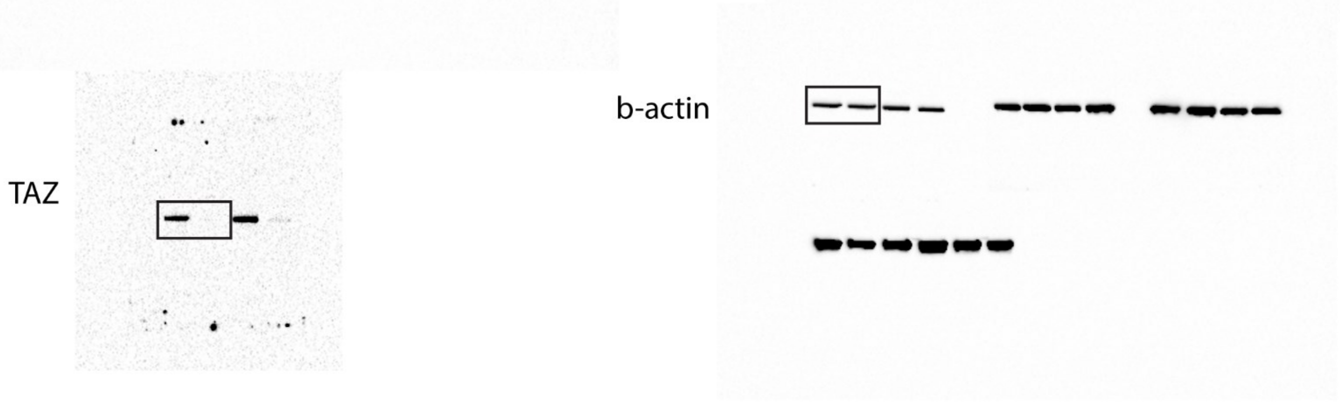

Figure 2F

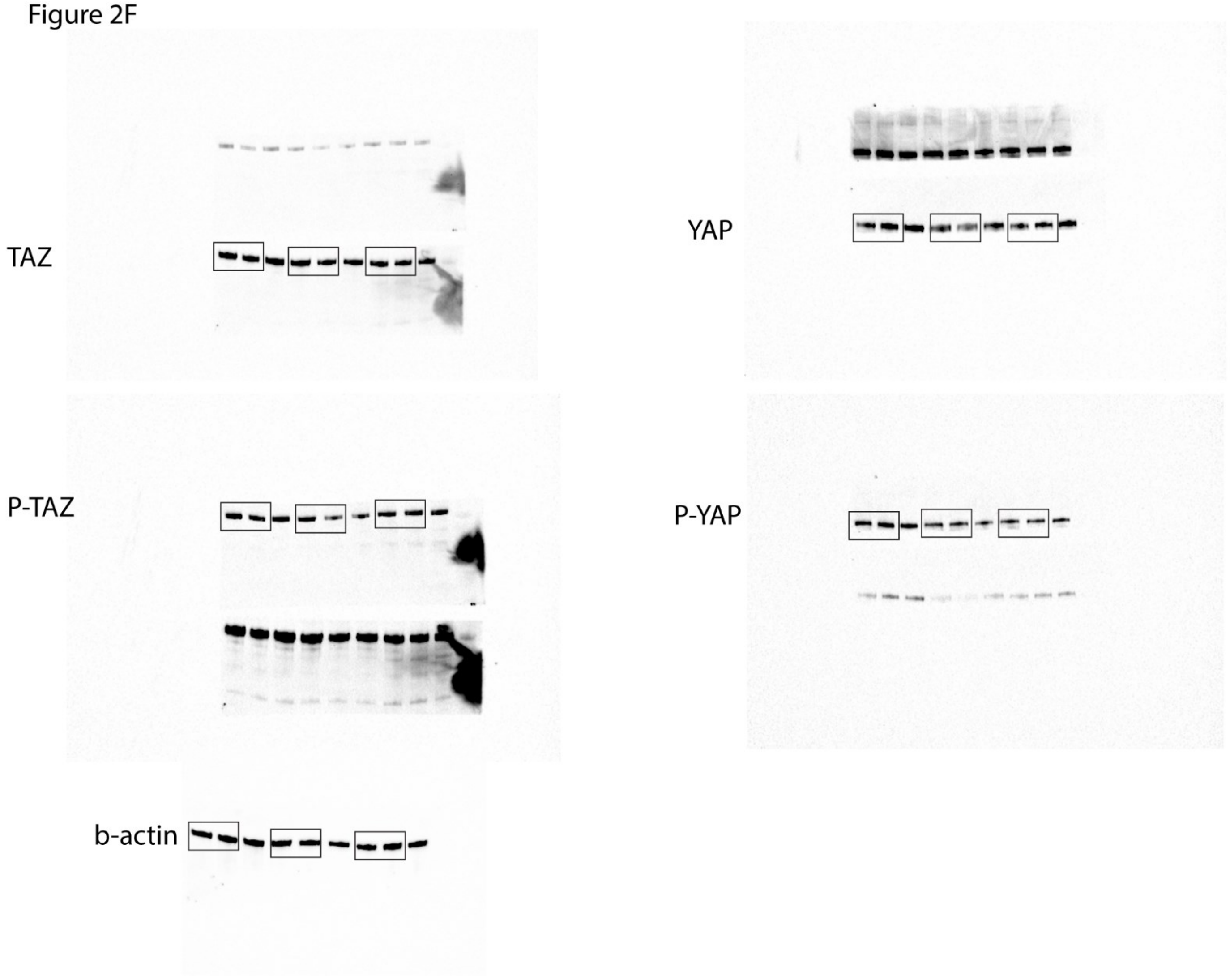

Figure 2G

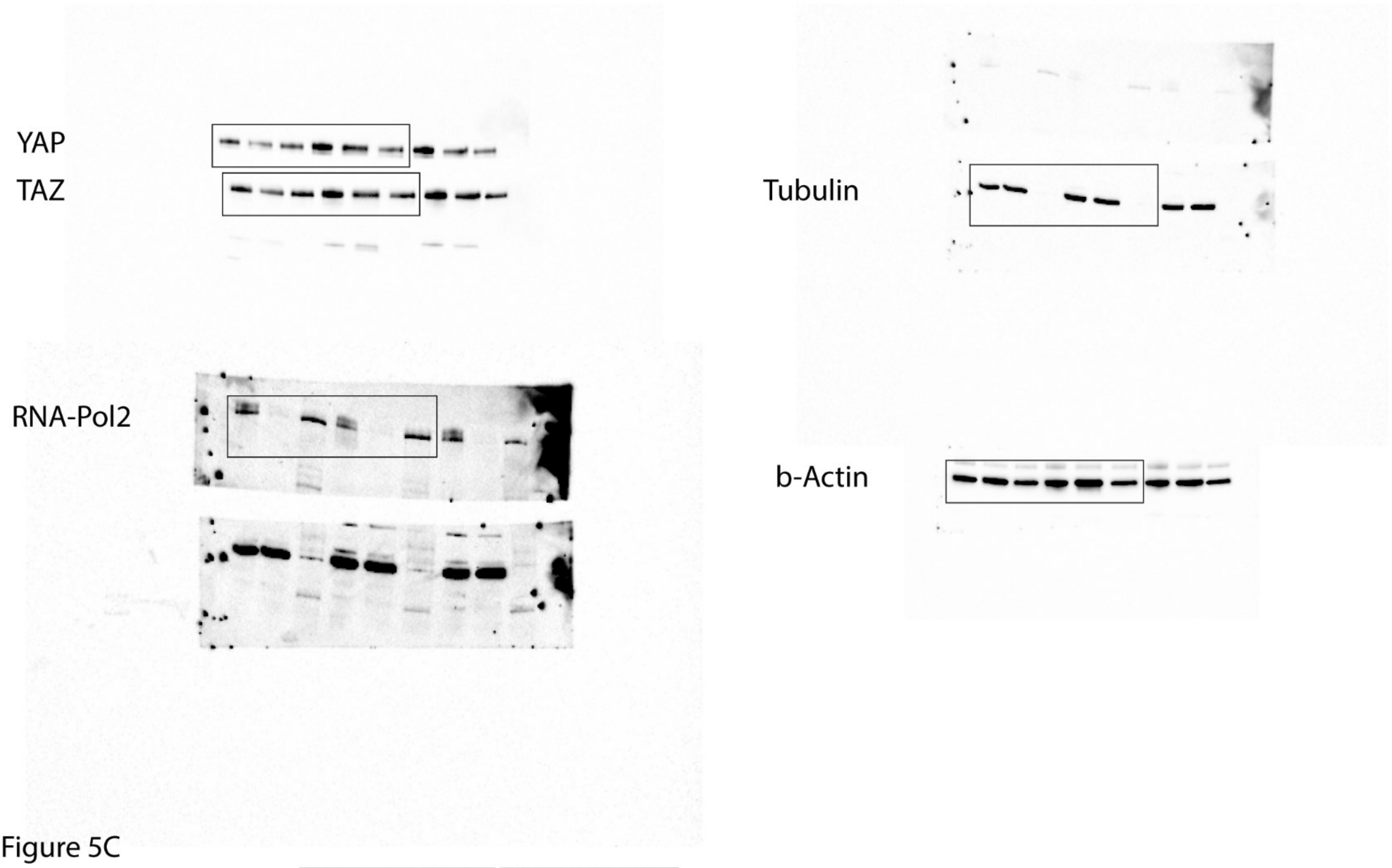

Figure 5C

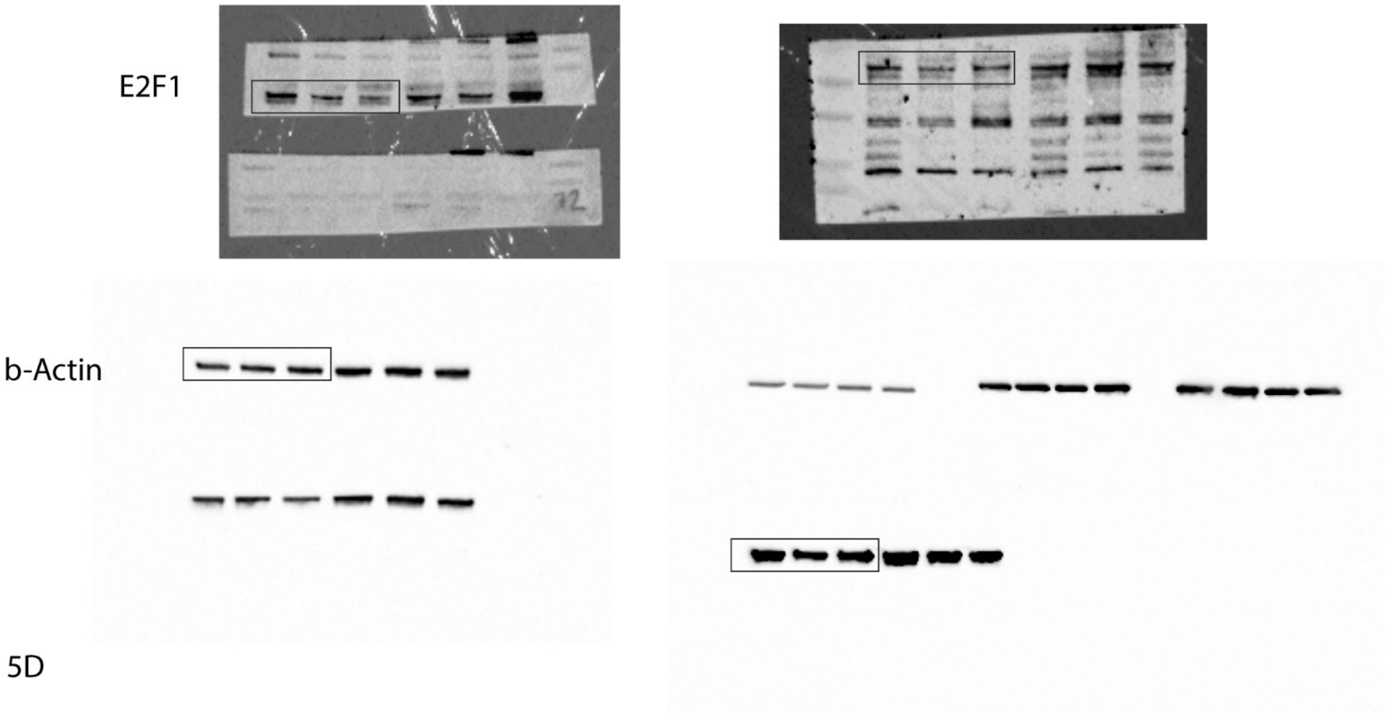

Figure 5D

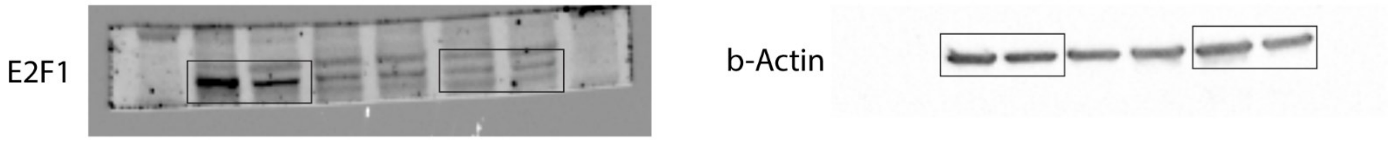

Figure 6A

E2F1

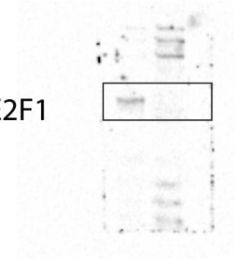

b-Actin

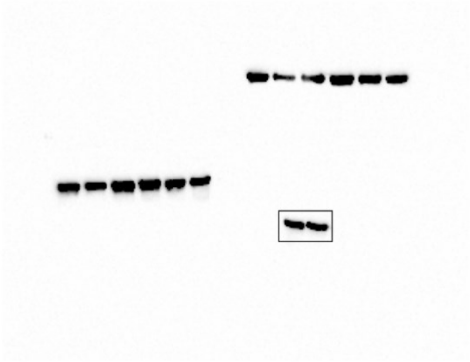

Figure 6B

E2F1

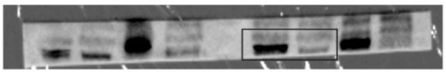

b-Actin

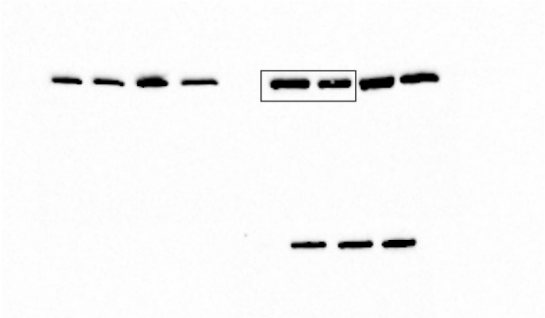

Figure 7A

EPHB2

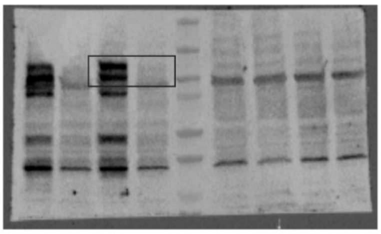

b-Actin

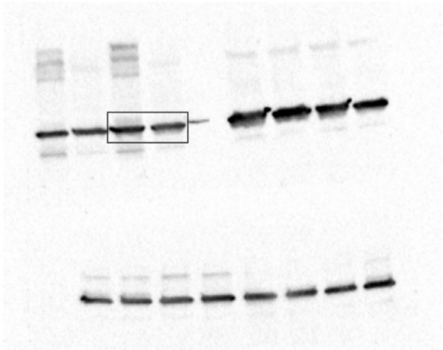

Figure 7B

EPHB2

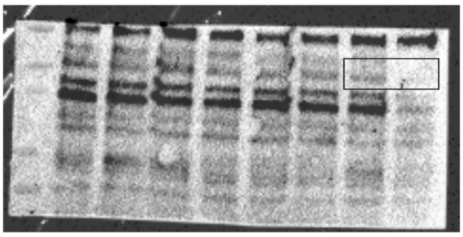

b-Actin

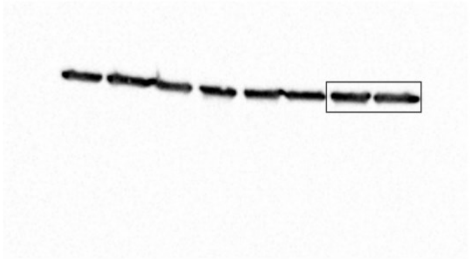

Supplementary Figure 2A

RNA-pol II

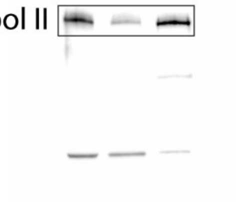

Tubulin

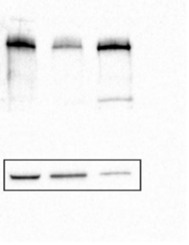

b-Actin

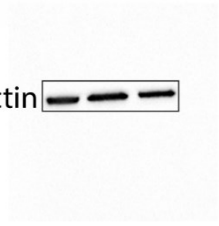

Supplementary Figure 2B

RNA-pol II

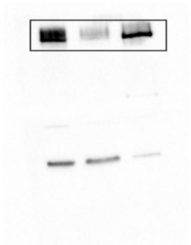

Tubulin

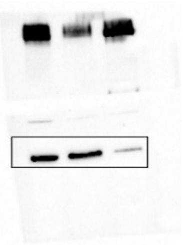

b-Actin

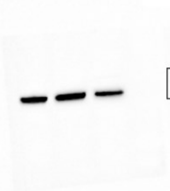

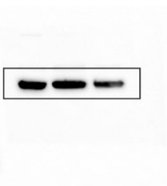

Supplementary Figure 3A

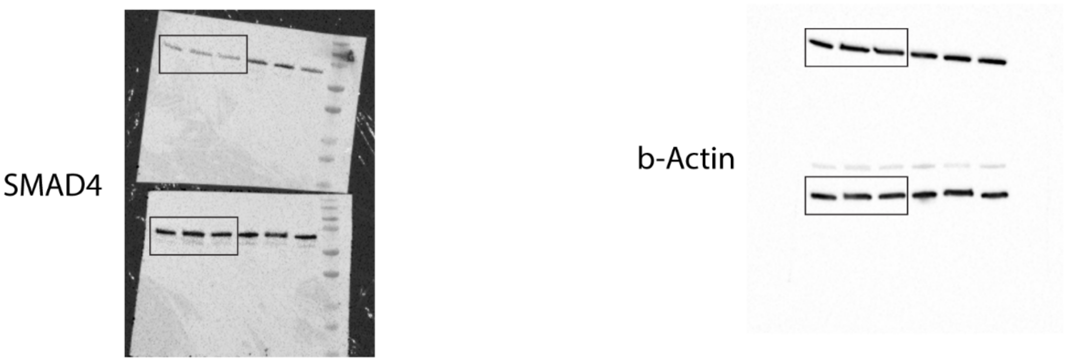

Supplementary Figure 3B

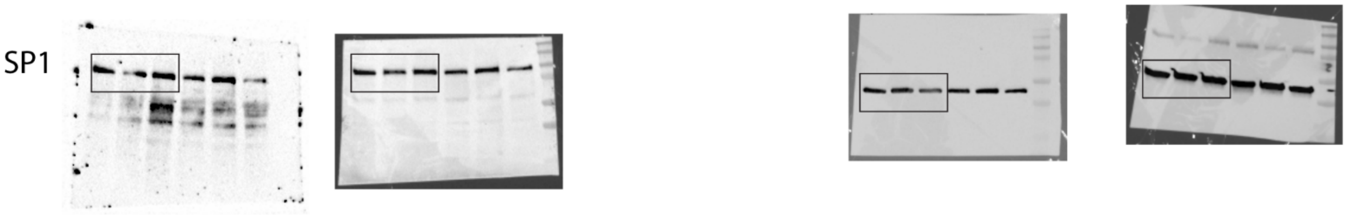

Supplementary Figure 3C

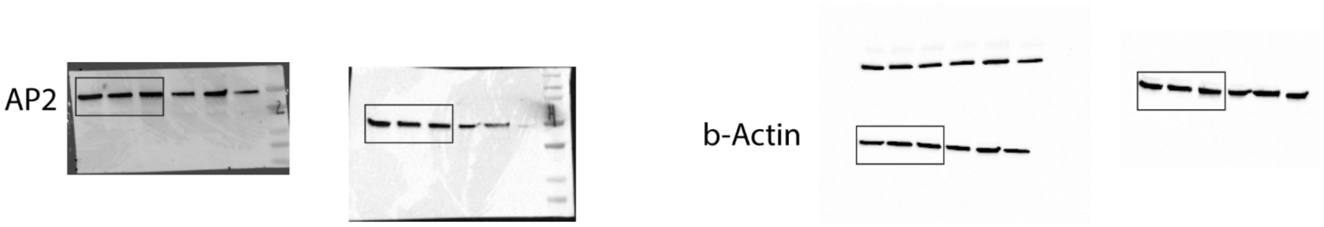

Supplementary Figure 3D

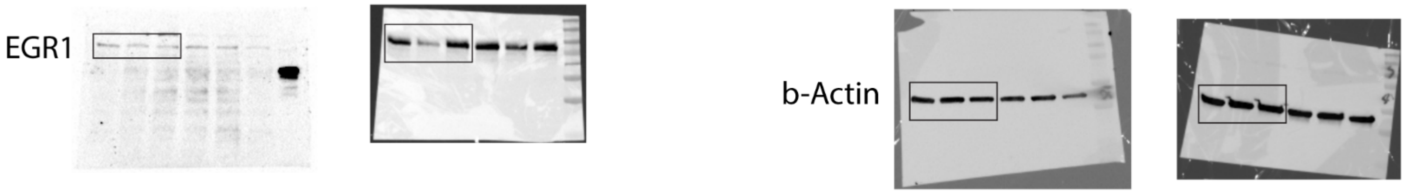

Supplementary Figure 3E

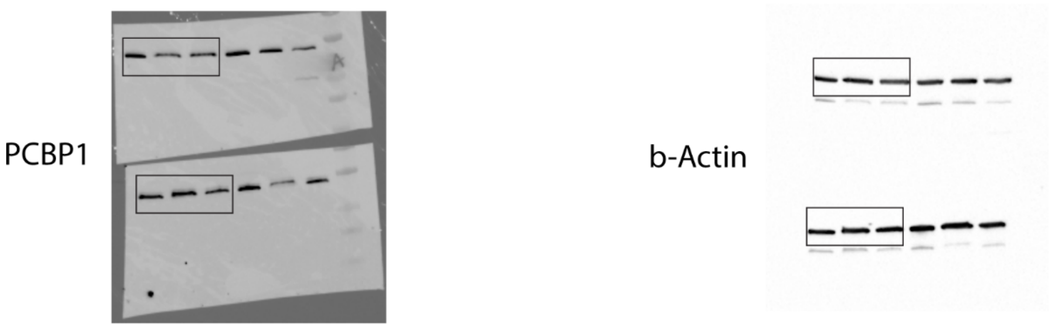

Supplement: Supplementary file 4 — Original data files [file 41419_2023_6277_MOESM4_ESM.pdf]
